# Creating an Equitable and Fair Experience for Psychiatry Core Trainees Completing the Mock CASC in Kent and Medway Mental Health NHS Trust

**DOI:** 10.1192/bjo.2026.11287

**Published:** 2026-06-30

**Authors:** Hanna Mansi, Rachel Rice

**Affiliations:** Kent and Medway Mental Health NHS Trust, Kent, United Kingdom

## Abstract

**Aims:**

The aim of our quality improvement project was to create an equitable and fair experience for psychiatry core trainees in Kent and Medway Mental Health NHS Trust (KMMH) who wanted to take part in our trust mock Clinical Assessment of Skills (CASC). This could be achieved by increasing candidate numbers and incorporating a quality assurance process.

KMMH runs a twice yearly mock CASC for resident psychiatry doctors. We mirror the Royal College of Psychiatrists(RCPsych) CASC exam as closely as we can with 16 stations, experienced actors and examiners in each station. We wanted to improve the quality of our mock CASC by aligning with RCPsych standardisation and increasing trainee satisfaction. Potential consequences of a poorly delivered training experience could result in trainees seeking training opportunities elsewhere, which has ramifications on building a sustainable and skilled workforce.

**Methods:**

We sought stakeholder opinion (examiners, candidates, senior medical education staff). We gathered current performance data: examiners requested marking guidance, there was a 56% discrepancy in second marking of the same candidate and only 56% of candidates were satisfied with the current mock CASC provision. We completed a root cause analysis by creating a fishbone and affinity diagram. The main themes identified were: lack of candidate opportunities, lack of examiner marking guidelines and marking inconsistency. Countermeasures were constructed from stakeholders, bench marking and best practice. A pick chart was used to identify the most beneficial and achievable countermeasures which were then implemented: double candidate places, reduce examiner bias by providing training, standardise marking through guidance and standardisations process. We created standardised marking guidance for each station which was provided to examiners in subsequent mock CASCs.

**Results:**

100% of examiners felt confident marking stations. Examiner marking consistency also improved from 44% to 47%. 100% of examiners were satisfied with the marking guidance given (previously was 50%). 100% of candidates (n=21) were satisfied with the mock CASC and found it beneficial for their learning, an increase from 56% prior to ourinterventions.

**Conclusion:**

Qualitative and quantitative data showed an improvement in trainee satisfaction and fairness of mock CASC with improved marking consistency. This provides evidence to embed standardisation process into future mock CASCs.

